# Supplementary material for: Far-Red Light Effects on Lettuce Growth and Morphology in Indoor Production Are Cultivar Specific
Source: Plants (Basel). 2022 Oct 14;11(20):2714. doi: 10.3390/plants11202714 (PMC9611250; doi:10.3390/plants11202714)
Supplement: Supplementary file 1 [file plants-11-02714-s001.zip › plants-1948317-Table S1.pdf]

Table S1. Details of light spectrums used in this study, including different metrics that quantify far-red light in spectrum.

Far-red *PFD* (700-750 nm) (μmol·m^{-2}·s^{-1})	*PPFD* (400-700 nm) (μmol·m^{-2}·s^{-1})	Total *PFD* (400-750 nm) (μmol·m^{-2}·s^{-1})	R:FR ratio ($\frac{600-700\ nm}{700-750\ nm}$)	FR fraction ($\frac{700-750\ nm}{400-750\ nm}$)
5.3	193.0	198.3	14.4	2.7%
5.6	197.2	202.9	13.8	2.8%
5.8	204.1	209.9	14.0	2.7%
11.5	194.0	205.5	6.7	5.6%
14.9	207.0	221.9	5.5	6.7%
15.5	204.9	220.4	5.2	7.0%
22.8	226.0	248.8	3.9	9.2%
34.3	195.9	230.2	2.3	14.9%
35.3	211.4	246.7	2.4	14.3%
36.1	219.7	255.8	2.4	14.1%
43.7	209.5	253.1	1.9	17.3%
51.2	207.8	259.1	1.6	19.8%
51.9	213.0	264.9	1.6	19.6%
53.3	194.0	247.3	1.4	21.6%
56.4	207.3	263.7	1.5	21.4%
56.9	205.0	262.0	1.4	21.7%
61.6	178.5	240.1	1.1	25.6%
75.9	209.1	285.1	1.1	26.6%
