# Supplementary figures and images for: Far-Red Light Effects on Lettuce Growth and Morphology in Indoor Production Are Cultivar Specific
Source: Plants (Basel). 2022 Oct 14;11(20):2714. doi: 10.3390/plants11202714 (PMC9611250; doi:10.3390/plants11202714)

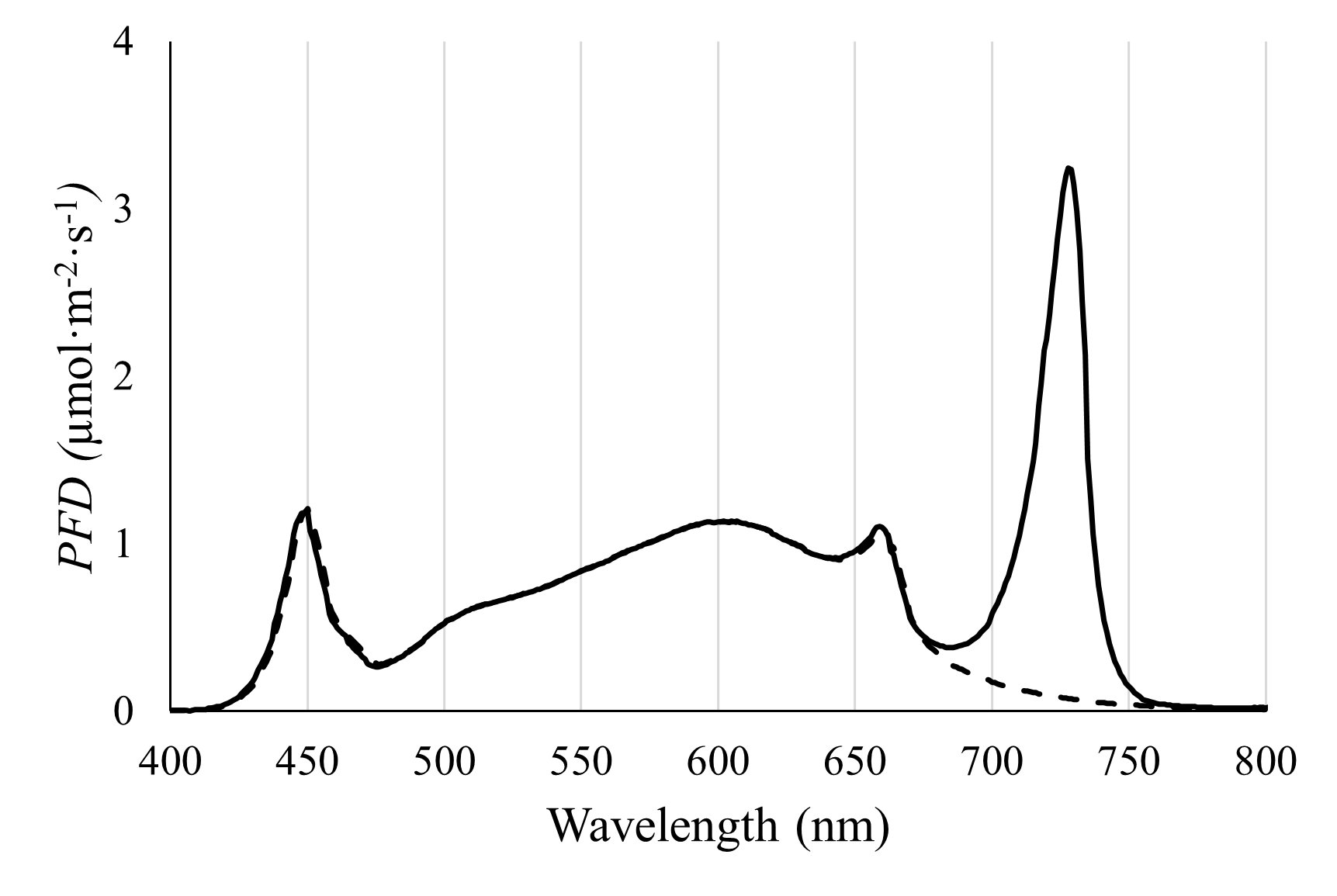

Supplement: Supplementary file 1 [file plants-11-02714-s001.zip › Figure S1.png]

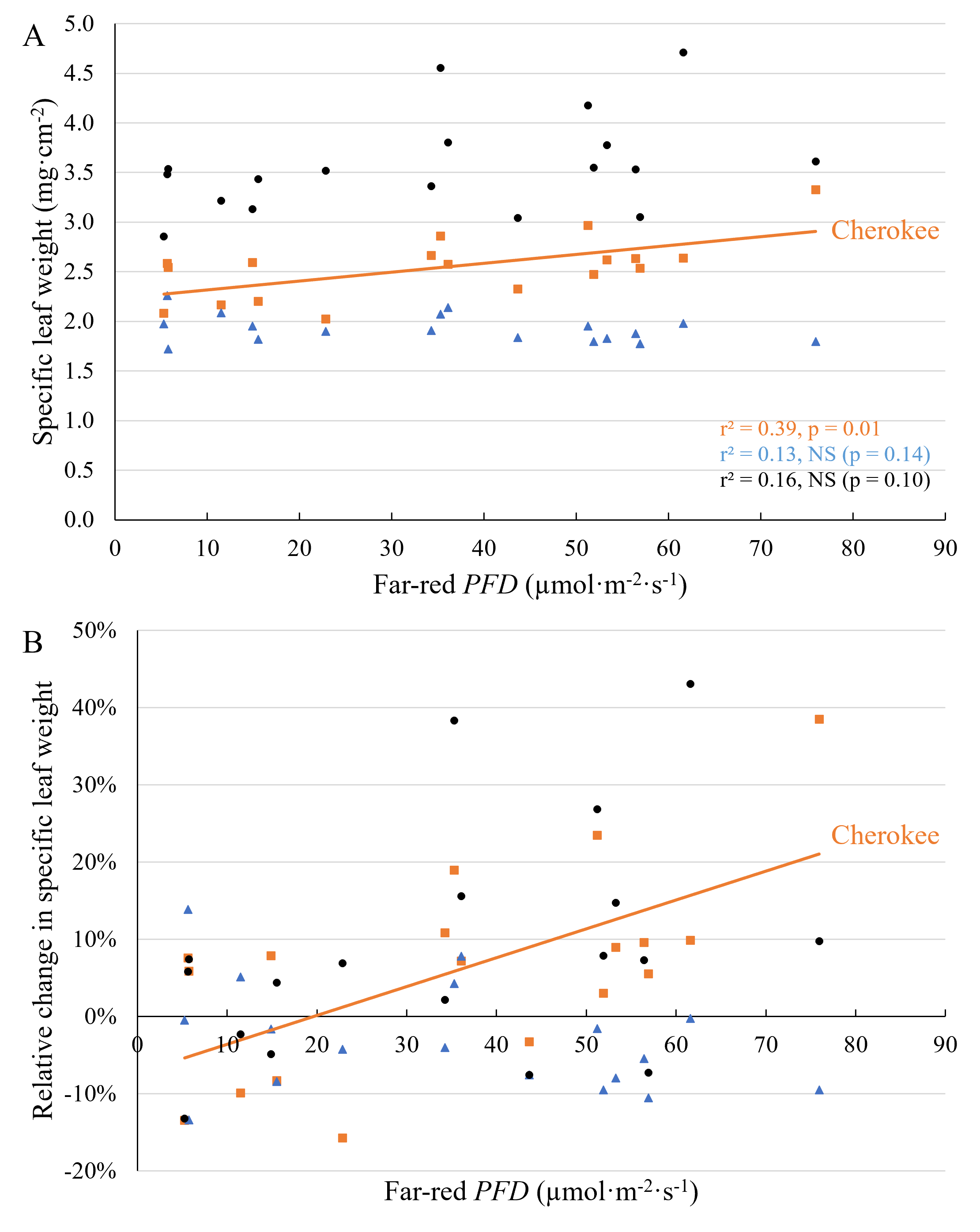

Supplement: Supplementary file 1 [file plants-11-02714-s001.zip › Figure S10.png]

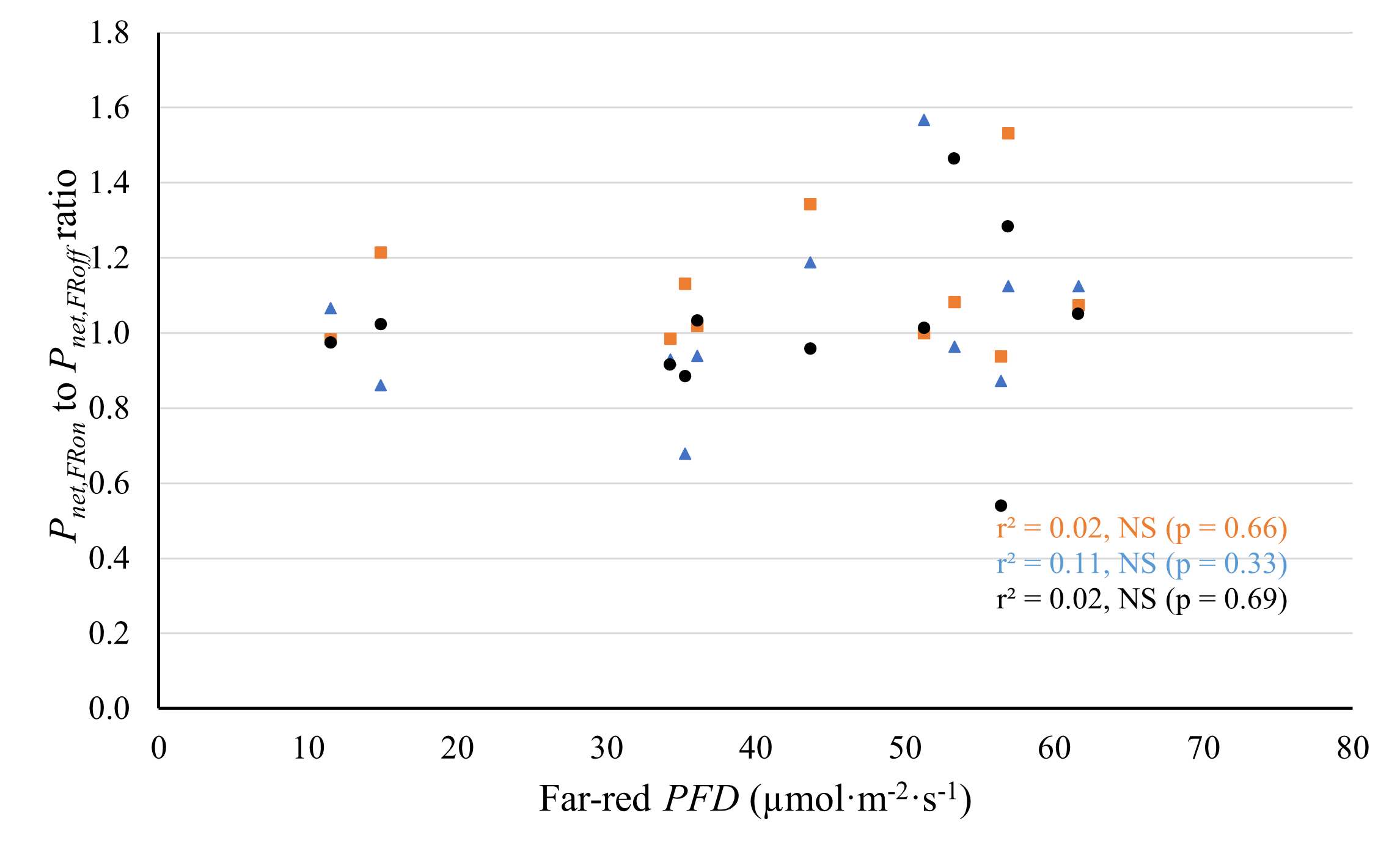

Supplement: Supplementary file 1 [file plants-11-02714-s001.zip › Figure S11.png]

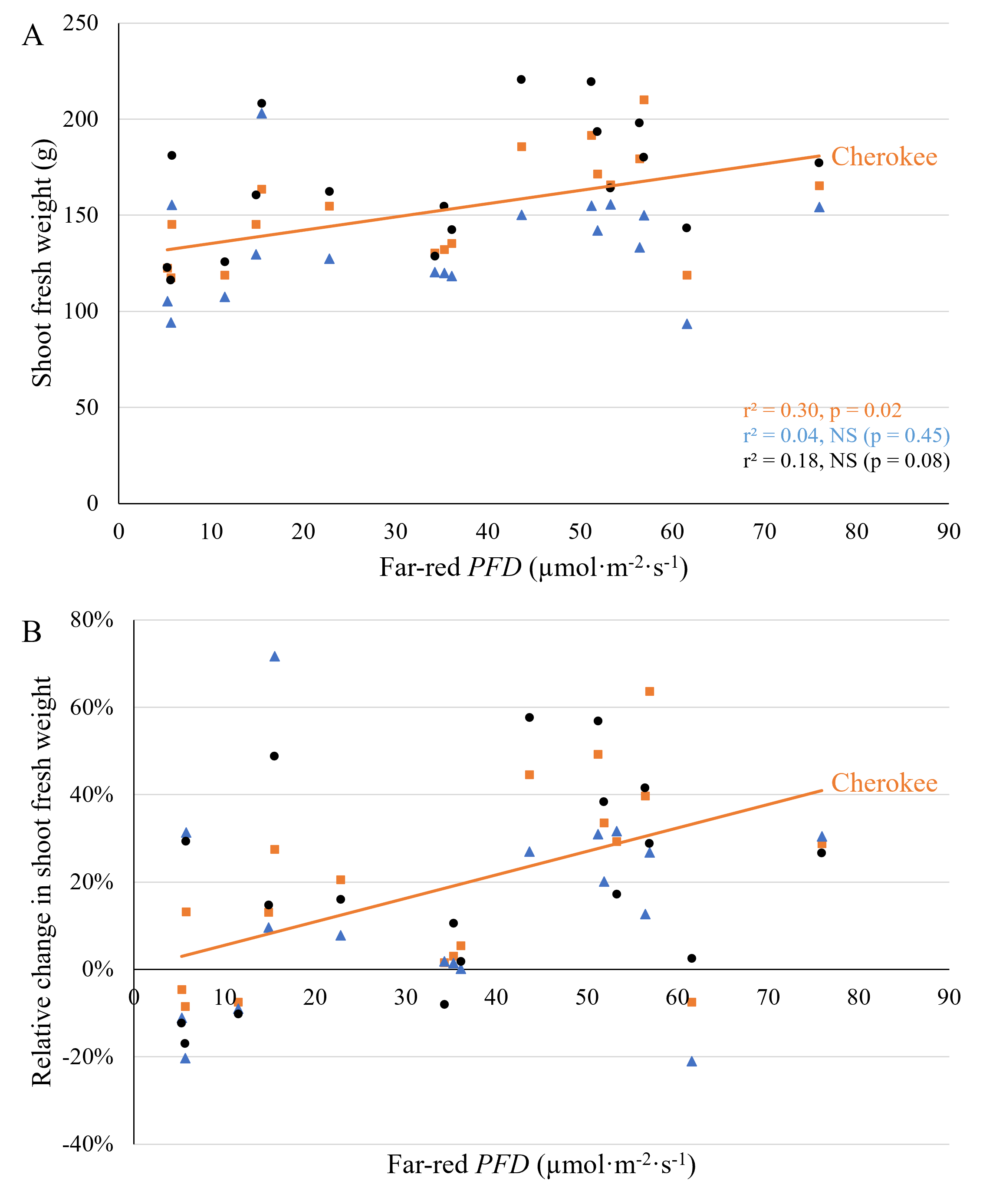

Supplement: Supplementary file 1 [file plants-11-02714-s001.zip › Figure S12.png]

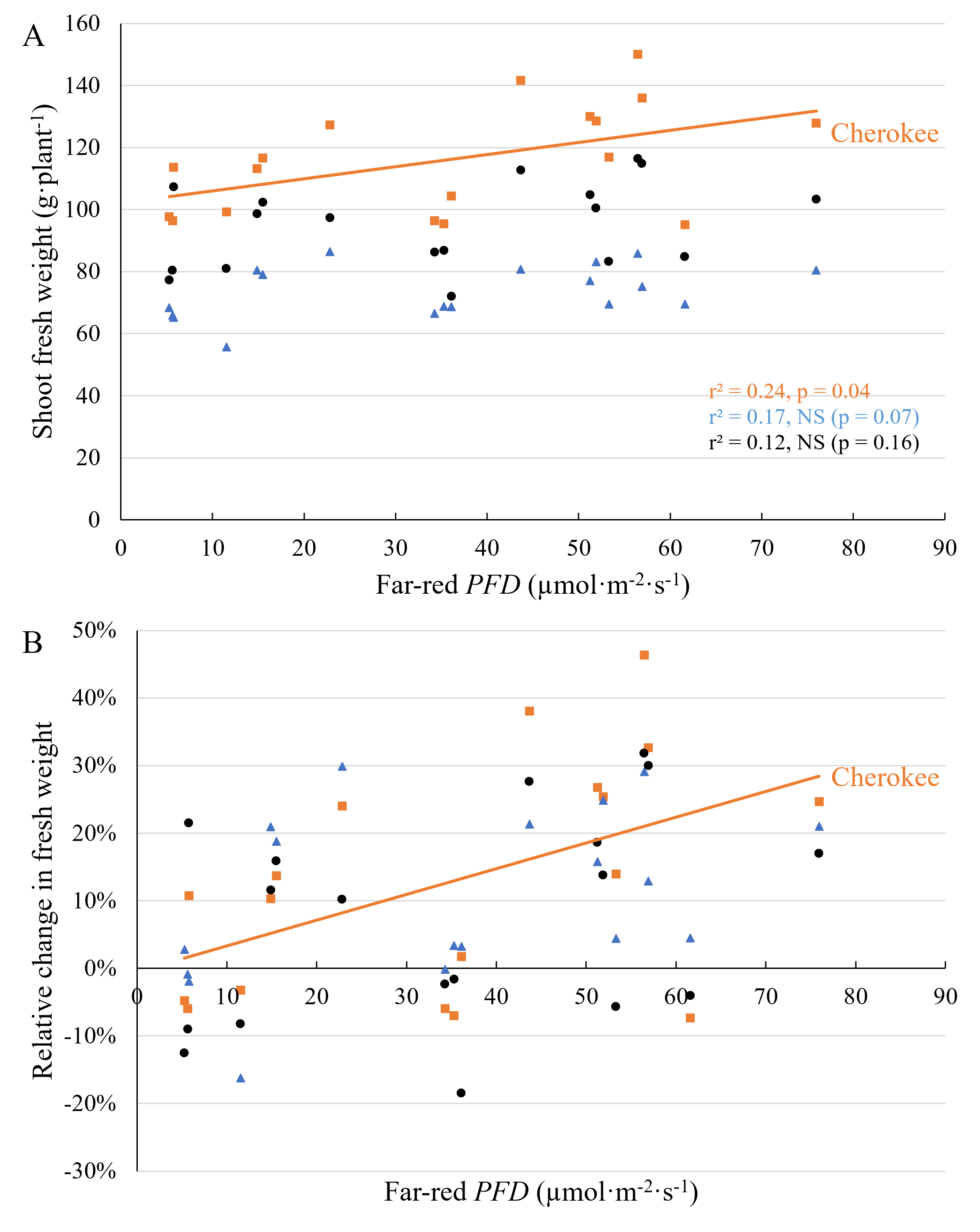

Supplement: Supplementary file 1 [file plants-11-02714-s001.zip › Figure S13.png]

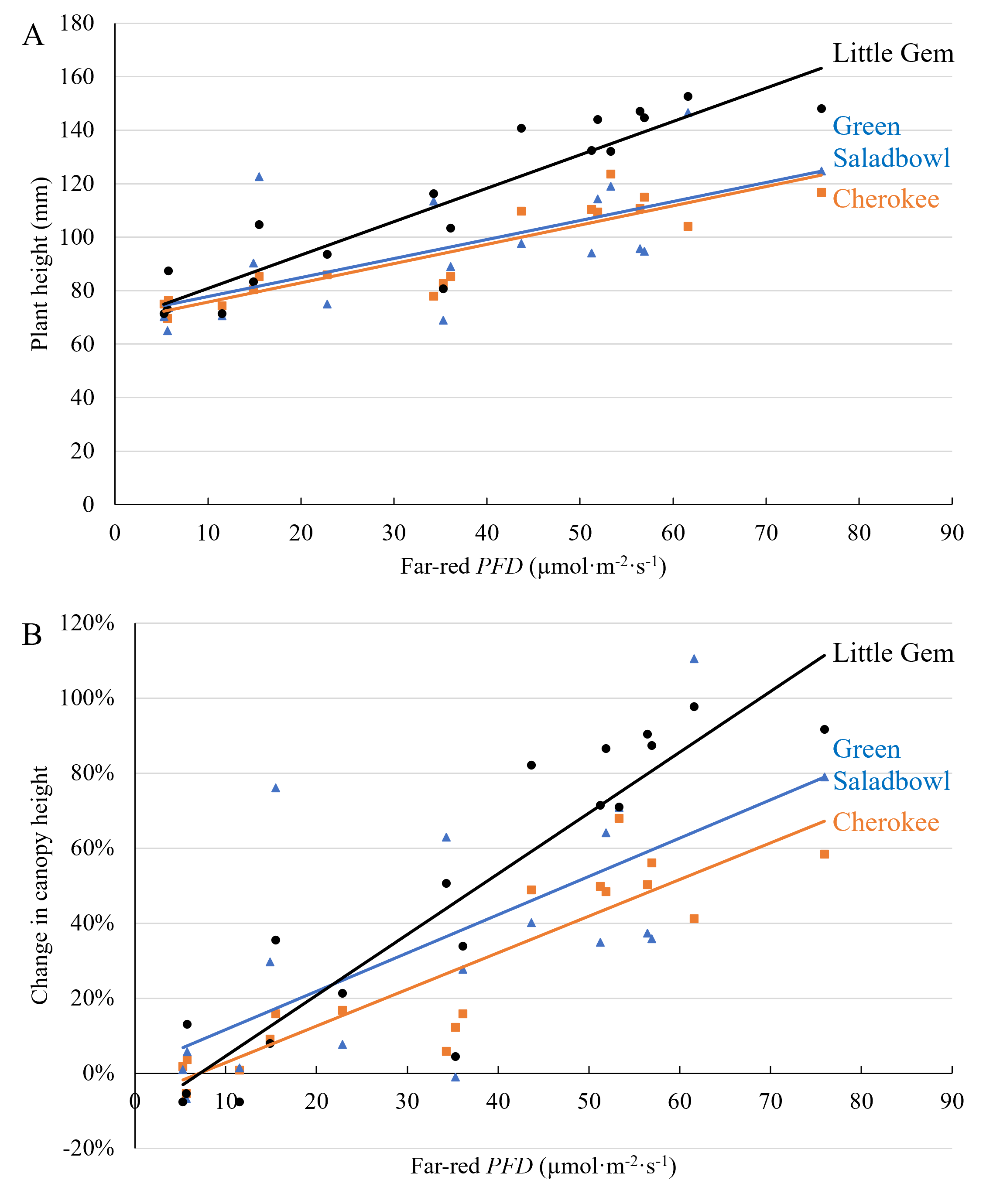

Supplement: Supplementary file 1 [file plants-11-02714-s001.zip › Figure S2.png]

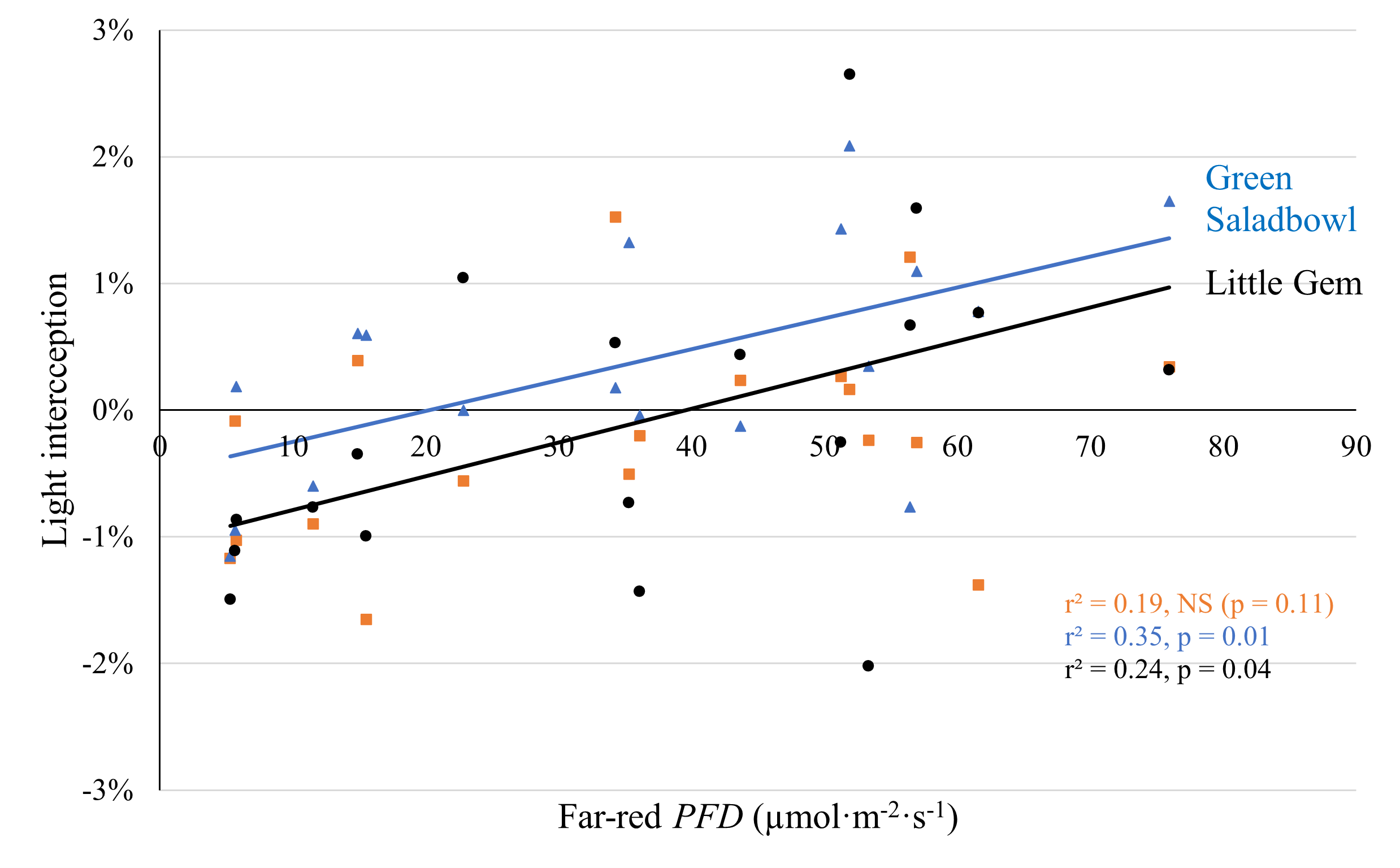

Supplement: Supplementary file 1 [file plants-11-02714-s001.zip › Figure S3.png]

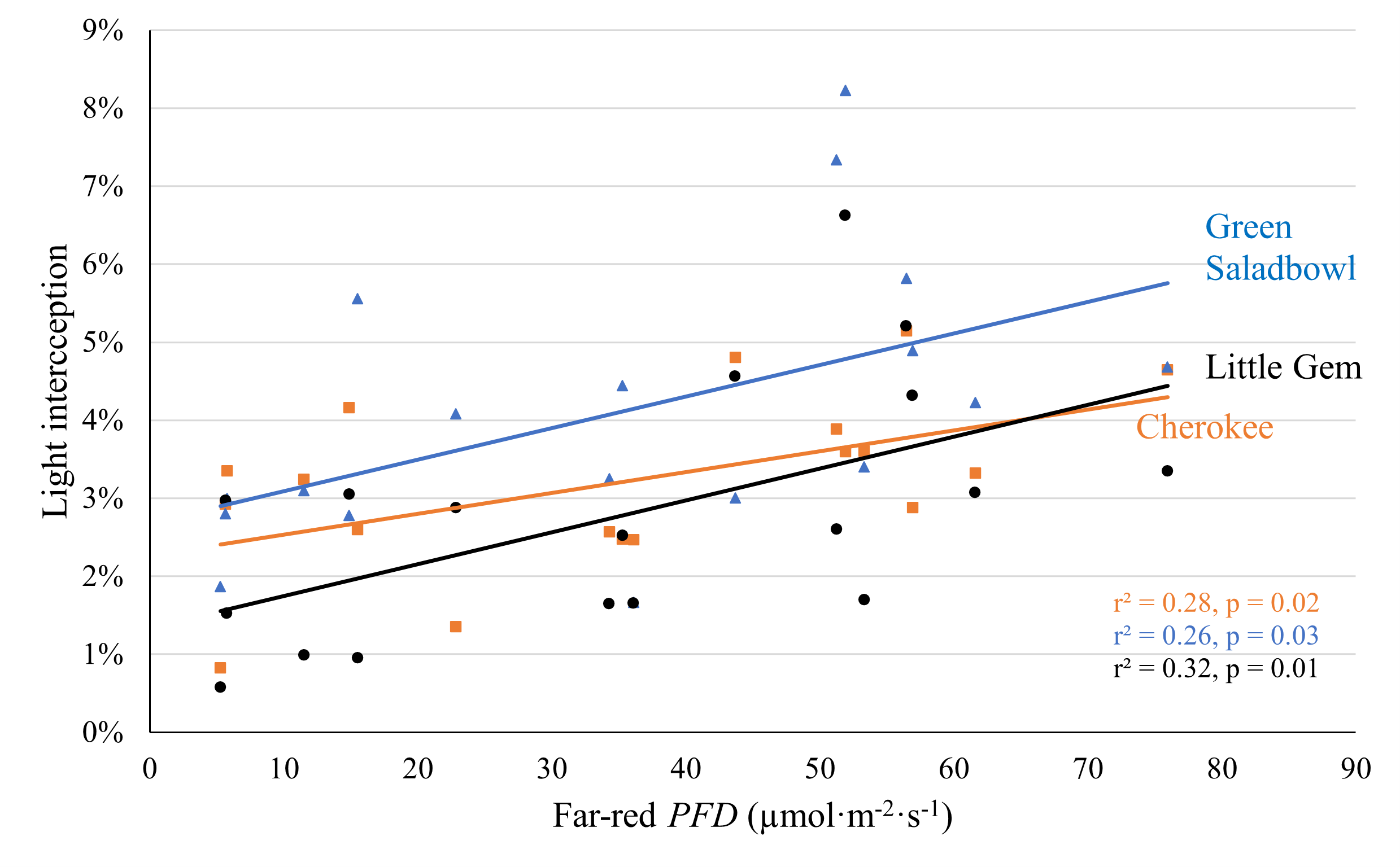

Supplement: Supplementary file 1 [file plants-11-02714-s001.zip › Figure S4.png]

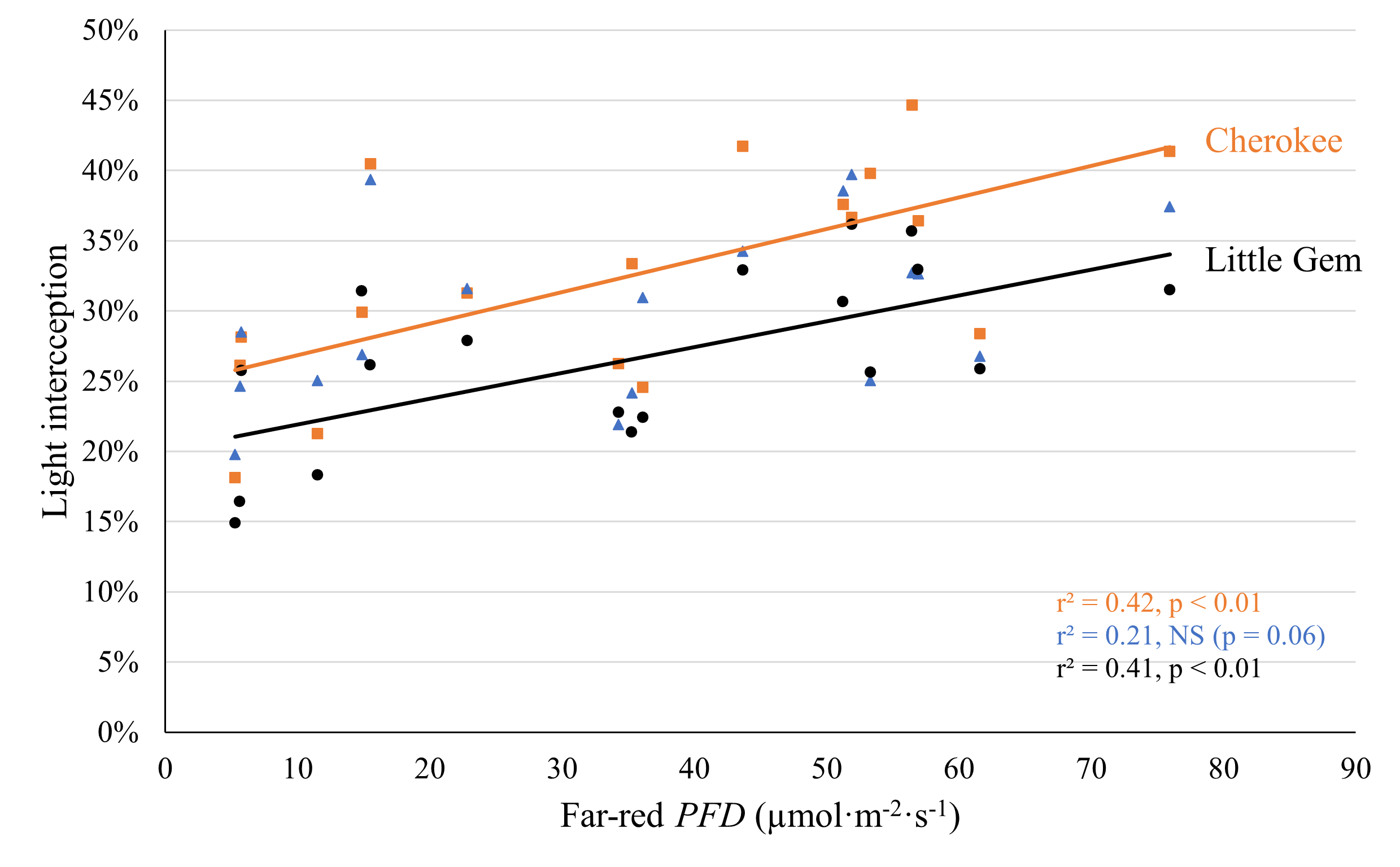

Supplement: Supplementary file 1 [file plants-11-02714-s001.zip › Figure S5.png]

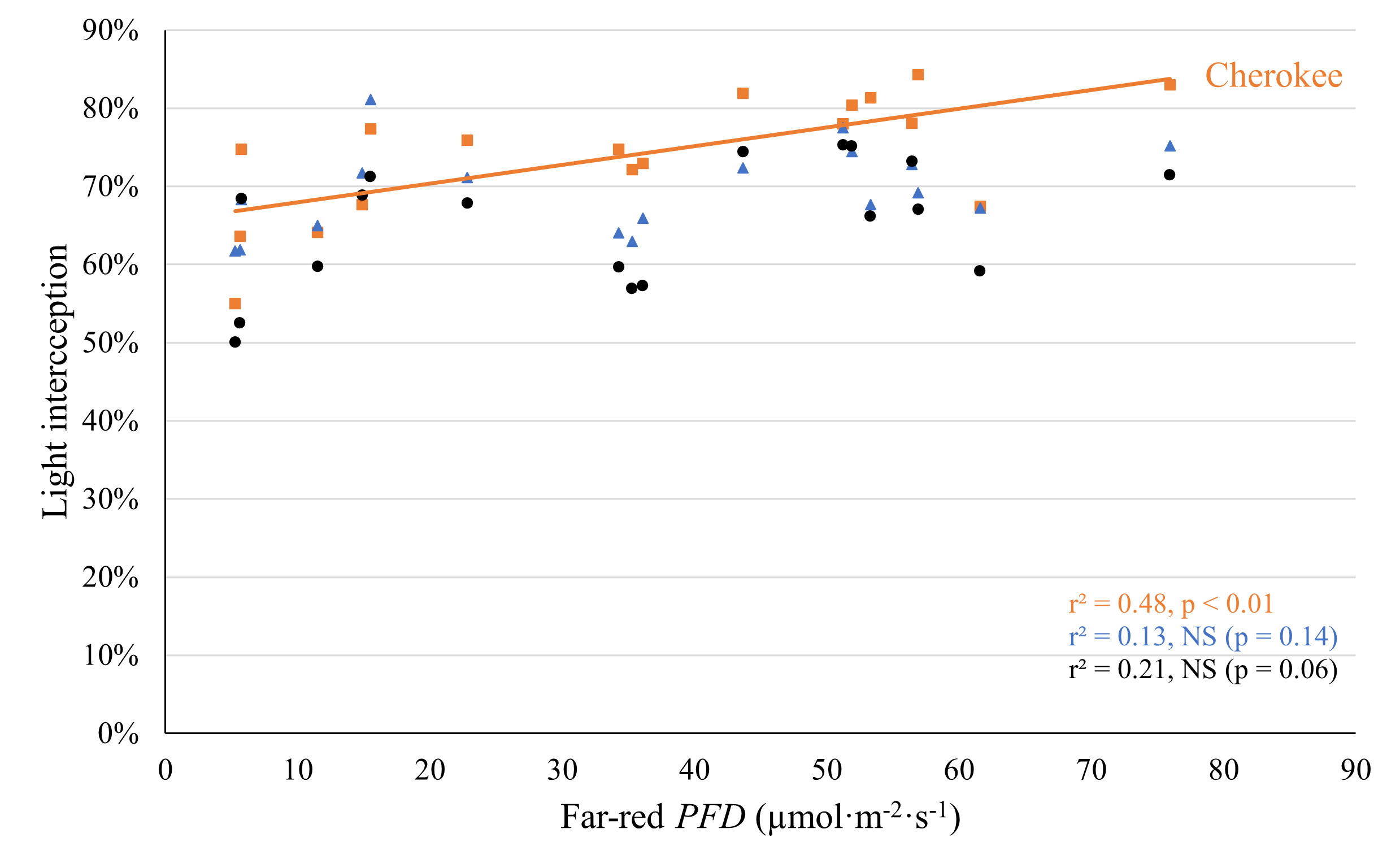

Supplement: Supplementary file 1 [file plants-11-02714-s001.zip › Figure S6.png]

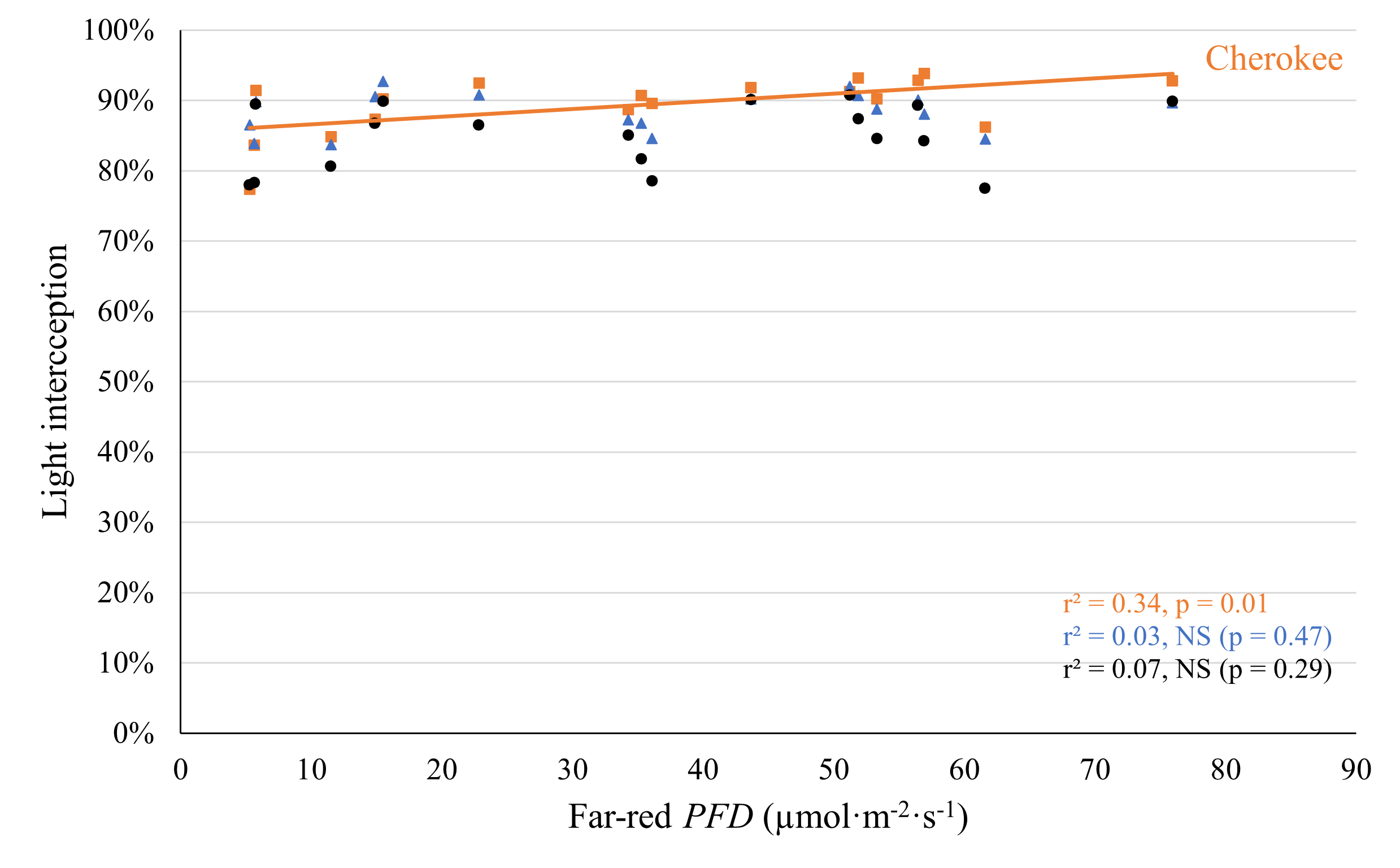

Supplement: Supplementary file 1 [file plants-11-02714-s001.zip › Figure S7.png]

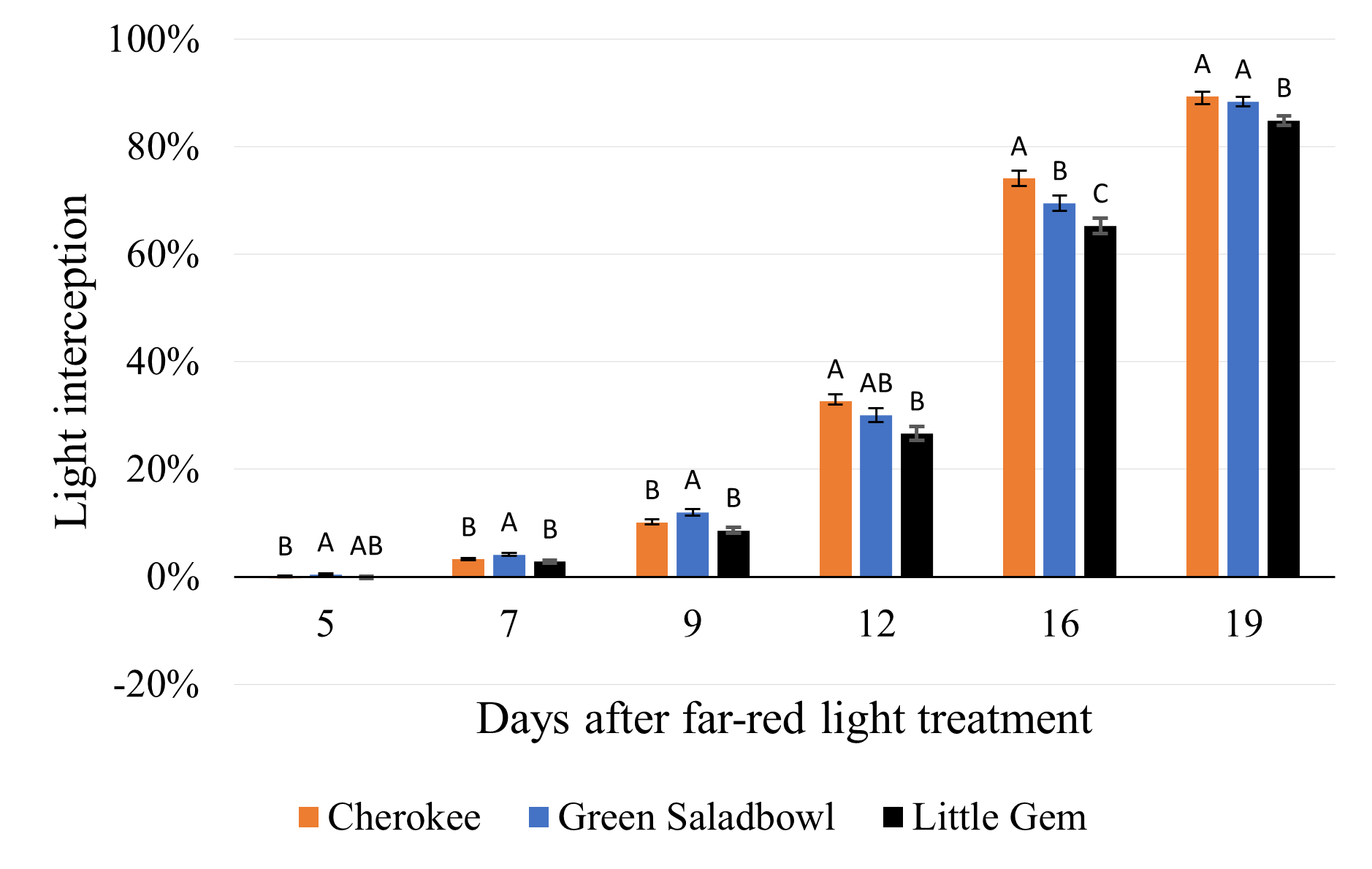

Supplement: Supplementary file 1 [file plants-11-02714-s001.zip › Figure S8.png]

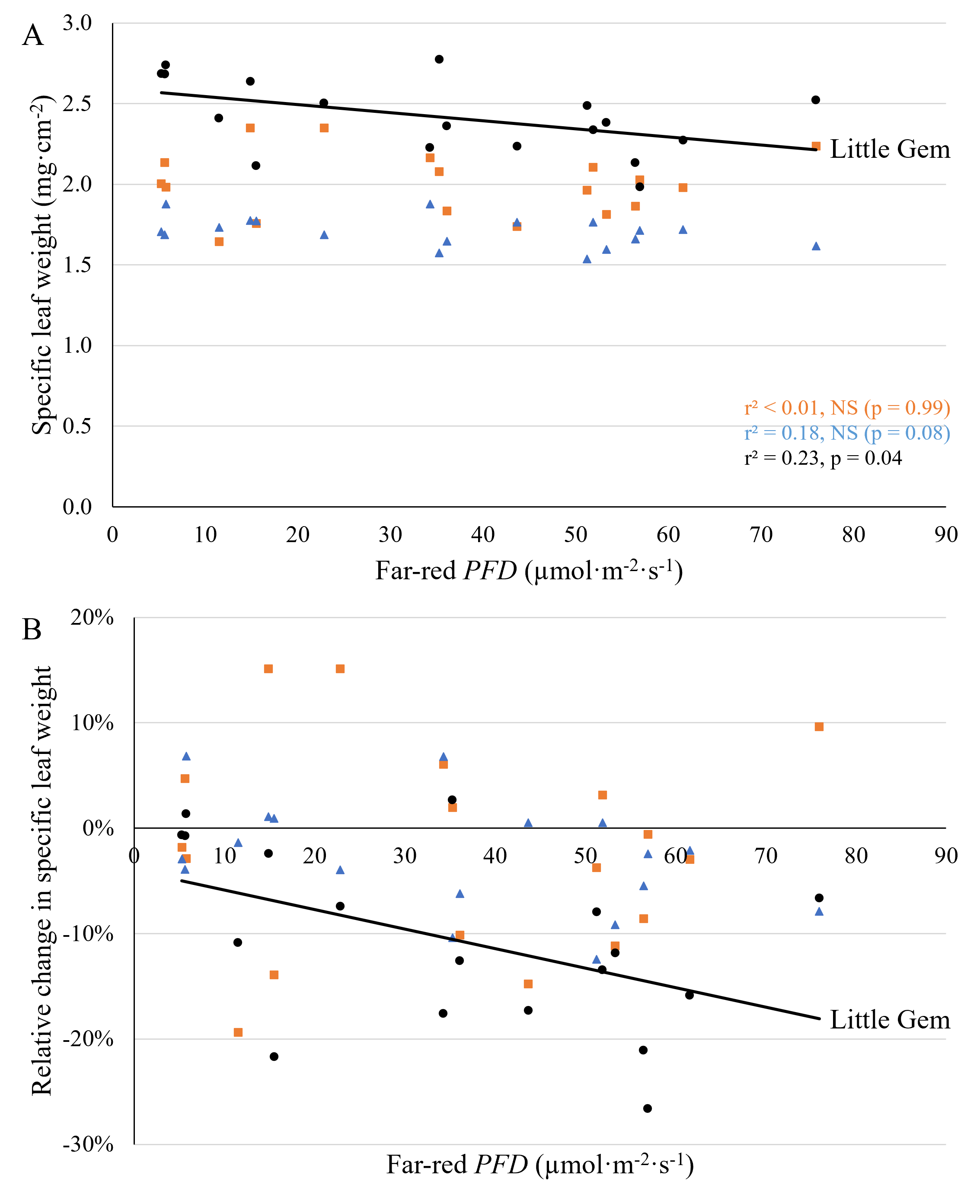

Supplement: Supplementary file 1 [file plants-11-02714-s001.zip › Figure S9.png]
